# The Action of Harrogate Water

**Published:** 1898-07-02

**Authors:** 


### THE ACTION OF HARROGATE WATER.

Anything that tends to lift the prescribing of mineral waters out of the regions of guesswork and empiricism is worthy of encouragement. It is, then, with much satisfaction that we note the very careful investigations which have lately been made by Dr. William Bain on the action of some of the well-known and much-prescribed waters of Harrogate, in regard to their power over the secretion of bile. By experiments made upon a man suffering from a permanent cutaneous biliary fistula, the action of the various waters, and of several other drugs, was carefully investigated. So far as the waters are concerned the result seem to show that the strong sulphur water from the Old Sulphur Spring, which generally acts as a mild laxative, has a very decided effect upon the secretion and composition of the bile, due to stimulation of the hepatic cells. No other drug which Dr. Bain experimented on produced such a striking increase in the quantity of both bile and bile solids as the Old Sulphur Spring. The Strong Montpelier Spring caused a diminution in the quantity of bile, in the solids, and bile salts. This water, therefore, is not a cholagogue. The Kissingen Spring produced a distinct increase in the quantity of the bile and a slight increase in the solids. The Chloride of Iron Spring, which is much used in the treatment of anæmia, has been generally thought to have the effect of diminishing the secretion of bile. This it seemed to do in the experiment, but the man was clearly not quite at the normal when the observations on this water were commenced, so that the results are not, perhaps, very conclusive. Carlsbad Mineral Water was also investigated, and the author says that he has no hesitation in saying that the high esteem in which this mineral water is held as a cholagogue is undoubtedly deserved. Hot water before breakfast, which by some is thought to equal all the Spas in Europe, was found to be disappointing, the quantity of bile being scarcely affected, while there was but a very slight increase in the solids. In regard to Harrogate waters, "it is a point of clinical interest that the relative empirical reputations of the Old Sulphur and the Strong Montpellier Springs are experimentally sustained."

